# Hepatocellular carcinoma tumor thrombus entering the right atrium treated with combining percutaneous and intravenous high-dose-rate brachytherapy: a case report

**DOI:** 10.1186/s42155-021-00259-x

**Published:** 2021-10-07

**Authors:** F. N. Fleckenstein, M. Jonczyk, E. Can, W. M. Lüdemann, L. Savic, T. Maleitzke, F. Krenzien, B. Gebauer

**Affiliations:** 1 Department of Diagnostic and Interventional Radiology, Charité - Universitätsmedizin Berlin, Berlin, Germany; 2 Berlin Institute of Health (BIH), Berlin, Germany; 3 Center for Musculoskeletal Surgery, Charité - Universitätsmedizin Berlin, Berlin, Germany; 4 Julius Wolff Institute. Charité - Universitätsmedizin Berlin, Berlin, Germany; 5 Department of Surgery, Campus Charité Mitte and Campus Virchow-Klinikum, Charité - Universitätsmedizin Berlin, Berlin, Germany

**Keywords:** Advanced HCC, BCLC, locoregional therapy, transvascular treatment, brachytherapy, atrial tumor invasion

## Abstract

The presented report describes a case of a Hepatocellular carcinoma (HCC) tumor thrombus (TT) infiltrating the inferior vena cava (IVC) and the right atrium (RA) in a 66-year old male patient who initially presented with TT related symptoms. CT-guided high-dose-rate brachytherapy (HDRBT) was performed for both, the intraparenchymal primary and the TT. A marked improvement of the tumor-related symptoms and shrinkage of the tumor mass were achieved six months after treatment initiation. The combination of intravascular and percutaneous HDRBT demonstrating a promising approach to palliate tumor-related symptoms in advanced HCC with macrovascular invasion.

## Introduction

Up to 6 % of all Hepatocellular carcinomas (HCC) cases present with a tumor thrombus (TT) in the inferior vena cava (IVC) (Gomes et al. 1992). Extensive growth of HCC into the IVC and even the right atrium (RA) is associated with a particularly poor prognosis. Eliminating the TT might therefore be an important target to improve hepatic function, thrombus associated symptoms and increase survival chances. In the past, several cases of successful treatment of a TT using locoregional therapy have been reported, yet none combined local with intravascular ablative approaches (Li et al. 2017, Gatti et al. 2019). In this report, we present the case of a patient with HCC accompanied by an extensive TT infiltrating the RA through the IVC treated with CT-guided high-dose-rate brachytherapy (HDRBT).

## Case

A 66-year-old male patient was diagnosed with a poorly differentiated HCC on the basis of a cryptogenic liver cirrhosis in November 2019. The tumor was biopsy proven and radiological tumor staging in January 2020 had resulted in T3a N0 M0.

**Fig. 1:**
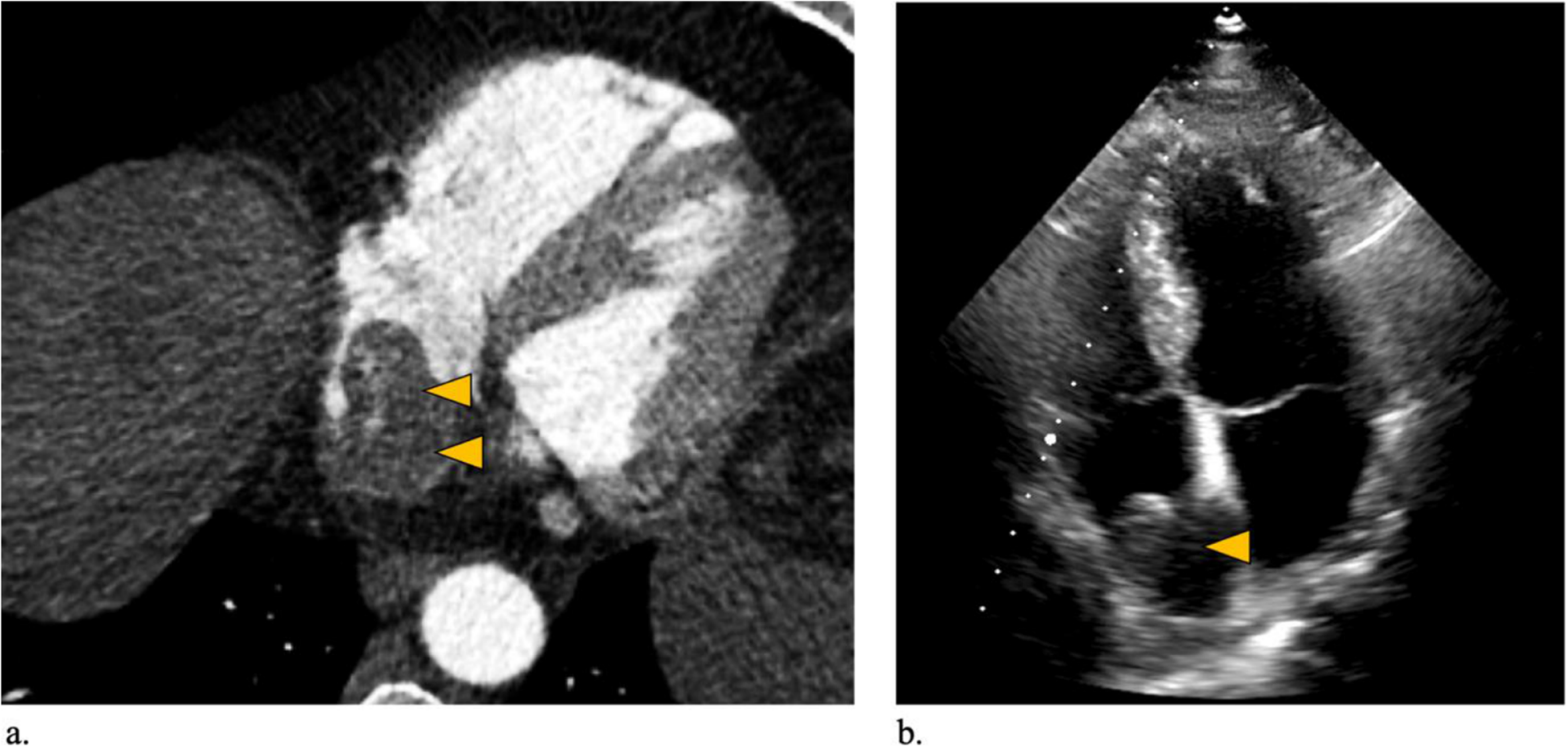
Pre-interventional imaging of the TT in the RA. (**A**) Pre-interventional axial CT-angiography showing extensive growth of the TT in the RA and confirming arterial enhancement in the TT. (**B**) Pre-interventional echocardiogram confirming a mass in the RA

The patient had a history of previous hepatitis A and E infection but tested negative for hepatitis B and C at the time of treatment initiation. Clinically, the patient presented with an Eastern Cooperative Oncology Group performance status (ECOG) of 2, mainly suffering from angina pectoris, tachycardia, leg edema as well as ascites. Echocardiogram confirmed a mass in the RA (Fig. 1 and attached video file). At initial presentation the cirrhosis was classified as Child Pugh A with an AFP of 3.2 ng/ml. Because of the advanced stage of disease, in the multidisciplinary tumor board, a decision was made to treat the patient with HDRBT in order to lower tumor burden and improve tumor-related symptoms in a palliative setting.

**Fig. 2:**
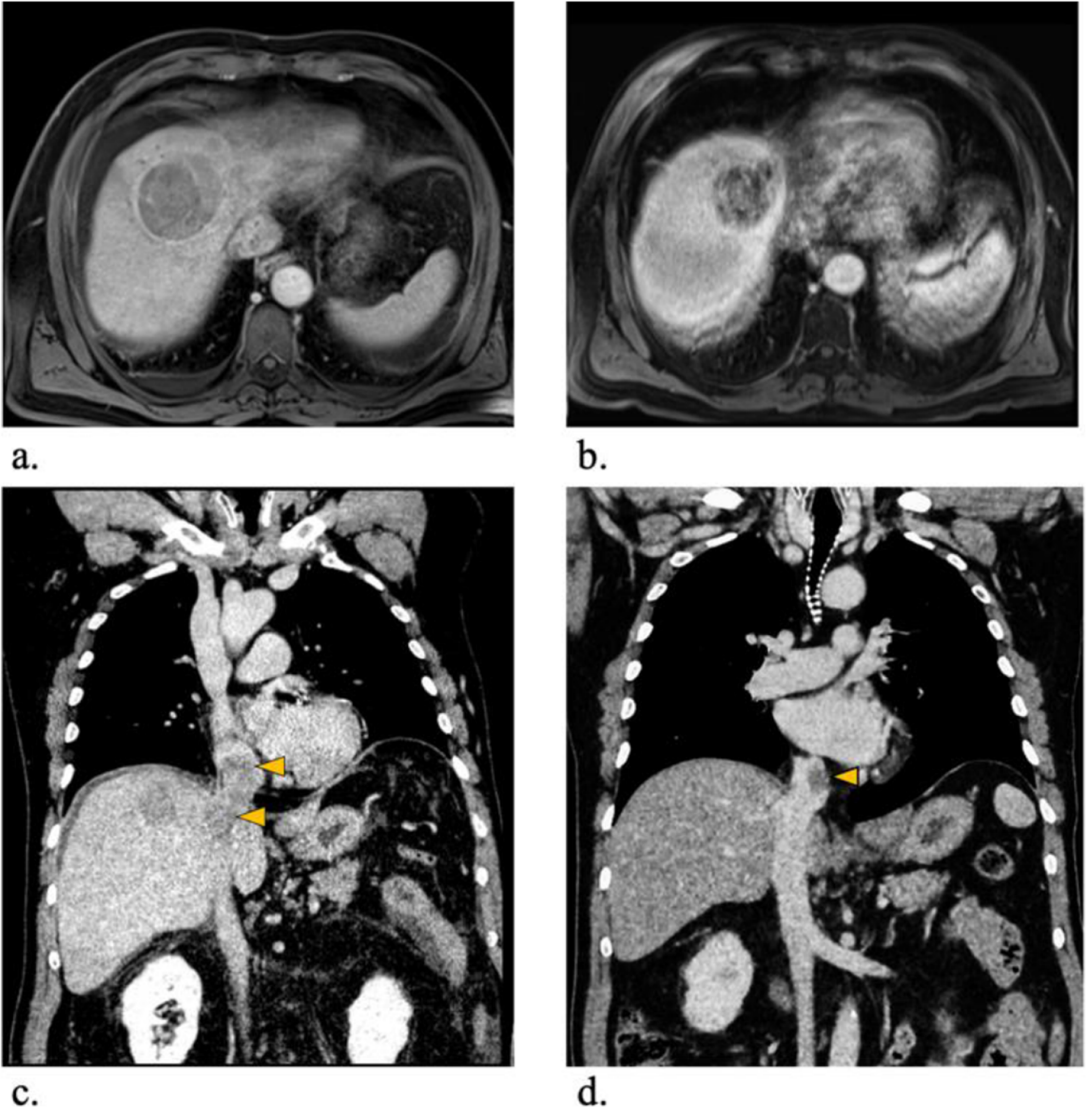
Pre- and post-interventional radiographic images. (**A**) Pre-interventional contrast enhanced MRI examination in the hepatobiliary excretion phase showing a large tumor lesion in segment IV/VIII in transversal plane. (**B**) six-months post-interventional MRI showing good radiological response to treatment using the same imaging technique. (**C**) Pre-interventional contrast enhanced CT examination confirms the presence of a TT entering the middle liver vein and extending through the IVC into the RA of the heart in the coronal plane. (**D**) six-months post-interventional CT showing marked shrinkage of the TT improving blood flow to the heart using the same imaging technique

In the initial contrast-enhanced magnetic resonance imaging (MRI) examination of the liver using gadoxetic acid (Gd-EOB-DTPA, Primovist®; Bayer, Berlin, Germany) the patient showed a 64 × 58 mm tumor in liver segment V/VIII with a satellite lesion of 16 × 11 mm in direct proximity. Both lesions showed arterial hyperenhancement, defects in the hepatobiliary excretion phase as well as diffusion restrictions (Fig. 2A). Further, the tumor was accompanied by a TT entering the middle hepatic vein extending through the IVC up into the RA of the heart measuring 5 cm in maximum axial diameter (Fig. 2C). Mild perisplenic and -hepatic ascites was present at initial presentation.

**Fig. 3:**
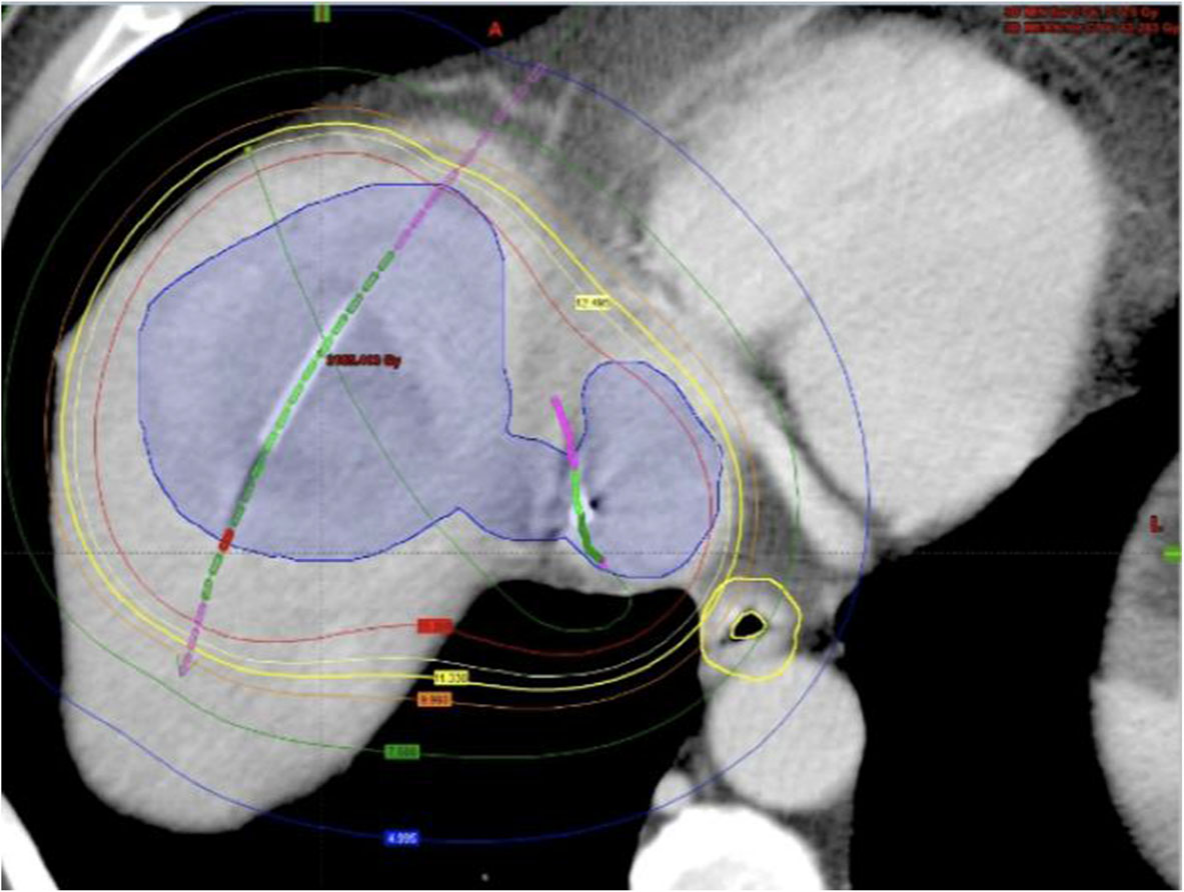
Peri-interventional 3D-irradiation plan. Contrast-enhanced planning computed tomography (CT) after CT-guided positioning of two afterloading catheters. Orange marker shows transcutaneous catheter 1, green marker shows transfemoral catheter 2. Visible tumour borders were defined as the clinical target volume (CTV) (blue line). Dose distribution was adjusted by 3D-treatment planning. The planned minimal enclosing dose was 15 Gy (blue line). Isodose irradiation lines surround the CTV

The HDRBT intervention was performed by an operator with more than 20 years of experience in interventional radiology (B.G.). Briefly, the lesion was punctured under CT-guidance via a 17 G coaxial needle. An angiography sheath 6 F in diameter (Radiofocus™, Terumo, Japan) was then inserted over a stiff angiography guidewire (Amplatz™, Boston Scientific, Boston, MA). Finally, one afterloading catheter (Primed™, Halberstadt Medizintechnik GmbH, Halberstadt, Germany) was inserted into the tumor lesion in Seldinger’s technique (Ricke et al. 2004). A second catheter (5 FR. Gammamed™ Bronchial catheter, Varian, Palo Alto, USA) was then placed in the IVC via a transfemoral access with the tip at height of the TT. After catheter placement, a multi-phasic contrast-enhanced CT scan of the upper abdomen was acquired to confirm correct positioning of both catheters and for radiation planning (Fig. 3). Brachytherapy was performed as single-fraction irradiation in afterloading technique using an iridium-192 radiation source (VARIAN GammaMed Plus iX HDR, Varian, Palo Alto, USA) with a nominal activity of 10 Ci. Radiation dose was determined based on previously reported data from our institution. In a palliative situation we aim for a dose of 15 Gy covering the tumor edges. In the central parts of the tumor, doses of > 50 Gy are accepted (Ricke et al. 2004). The clinical target volume (CTV) was 247 ml, the intended dose of at least 15 Gy could be deployed in 90,6 % of the CTV. Effective irradiation time was 37 min and total procedure time was 1 h and 42 min. For sedation and pain management during catheter positioning and radiation, we applied 75 µg fentanyl (Fentanyl 0,5 mg, Rotexmedica GmbH, Trittau, Germany) and 1.5 mg midazolam (Midazolam 5 mg, HEXAL AG, Holzkirchen, Germany) intravenously as well as local anesthesia with lidocaine (Licain 1 % 10 mg, PUREN Pharma GmbH & Co. KG, Munich, Germany) subcutaneously prior to puncture.

In order to prevent secondary bleeding after removal of the percutaneous brachytherapy catheter, torpedo-shaped gelatin sponges (Gelfoam® absorbable gelatin sponge, USP, Pfizer, New York, USA) were inserted into the puncture tract. Because of radiation exposure of the esophagus and gastric wall, proton pump inhibitors were prescribed (Pantoprazole 40 mg, HEXAL AG, Holzkirchen, Germany) for eight weeks. To minimize the risk of pulmonary embolism, heparin prophylaxis was given once a day for six weeks (0.3 mg Fraxiparine®, Aspen Pharma Trading Limited, Dublin, Ireland).

The patient was discharged two days after intervention in a good clinical condition. No complications occurred in the post-interventional period. Contrast-enhanced CT and MRI scans were performed eight weeks and six months after the procedure. Of note, in the first follow-up eight weeks post-treatment no significant size reduction could be detected. In contrast, at six-month follow up a local tumor control was achieved with significant necrosis of the intrahepatic HCC lesion and shrinkage from 64 mm to 49 mm in largest diameter. The TT reduced from 54 mm to 25 mm in diameter (Fig. 2).

Clinically, the patient showed a general improvement of his condition with complete resolution of tachycardia, leg edema, and ascites along with an improvement of ECOG score from 2 to 1 and his liver function stabilized (bilirubin 1.3 mg/dL, INR 1.1, albumin 41 g/l, and PLT 185/nl). As of October 2020, after ten months of follow-up, the patient is clinically in a good condition without any systemic drug treatment.

## Discussion

This is the first report of a combined use of percutaneous and intravascular HDRBT to treat HCC with a TT to the IVC and RA demonstrating the feasibility, safety, and efficacy of this palliative treatment approach.

Despite multidisciplinary concepts and evolving therapeutic options, the management of advanced HCC remains challenging. Especially patients suffering from macrovascular tumor invasion show poor overall survival (OS) rates and optimal treatment remains undetermined. Tyrosine kinase inhibitors are recommended by major guidelines as the first-line therapy in case of macrovascular vessel invasion (Cornberg et al. 2019). However, the optimal treatment strategy for advanced HCC is yet to be defined. Compelling evidence on the superiority of either systemic or interventional/surgical approaches is not available due to the lack of high quality RCTs. Since HCC with TT in the IVC or RA has a high risk of pulmonary metastases, embolisms or occlusion of the tricuspid valve, interventional approaches might be an option in a palliative setting, even if complete tumor remission is not possible (Gomes et al. 1992, Saynak et al. 2007, Nagasue et al. 1984).

For cases with TT to the IVC or RA, the literature suggests that surgical therapy is an effective therapy with OS varying from 11 to 19 months. Given the complex nature of the surgery however, mortality and morbidity rates are high (Florman et al. 2009, Sakamoto et al. 2018). Especially in patients with extensive cirrhosis surgical treatment is often not an option. As other authors suggest, Radiotherapy might be a safe and effective option for the treatment of a TT in the IVC with good response rates up to 48 % (Pao et al. 2019).

Several, retrospective, observational studies that investigated the outcomes after radiofrequency ablation (RFA) of TT in the PV or IVC were published recently presenting safe and effective treatment results (Li et al. 2017, Gatti et al. 2019, Giorgio et al. 2009, Giorgio et al. 2014). With a low risk of complications and high OS rates HDRBT might be a suitable palliative treatment option in patients suffering from advanced HCC with tumor invasion into the IVC and/or RA. We chose HDRBT over RFA on the one hand because of a better accessibility of the long-shaped, floating TT via a transvenous catheter in the IVC and on the other hand because of the typical limitations of RFA. In general, RFA produces smaller ablation volumes and is subject to the heat-sink effect. Microwave ablation (MWA) is reported to be less prone to heat-sink effect. However, with frequencies up 2450 MHz an ablation near the heart holds the risk for peri- and post-interventional arrythmia (Mulier et al. 2002). HDRBT does not face these major limitations. Moreover, it does not require general anesthesia and is well tolerated by most HCC patients (Brinkhaus et al. 2014). Treatment volumes for a complex and large lesion can easily be shaped with multiple catheters in one session or the treatment can be repeated for stepwise approaches. HDRBT has become increasingly important in the management of primary and secondary liver tumors in centers around the world and has been validated by numerous studies to be safe and efficient (Ricke et al. 2004, Brinkhaus et al. 2014). One of the largest studies evaluated 98 patients treated in our institution with 212 unresectable HCC tumors (Collettini et al. 2015). Only 18 (8.5 %) of the tumor lesions showed local progression during their mean follow up of 23.1 months. Furthermore, no major complication was recorded in the first month after HDRBT, making it a very feasible and safe procedure.

Given the encouraging experience in this patient, further studies to evaluate the benefit of HDRBT in advanced HCC stages in a palliative treatment are currently being planned.

## Conclusions

In patients suffering from advanced HCC accompanied by symptomatic tumor extension into the IVC a combination of transvenous and percutaneous HDRBT might be a promising approach to palliate tumor-related symptoms in decompensated and advanced HCC stages. Larger scaled clinical trials will be necessary to confirm this preliminary report.

## Data Availability

All data generated or analysed during this study are included in this published article.
